# Expanding the concept of serotoninomics: perspectives for serotonin studies in the 20’s of the 21st century

**DOI:** 10.3389/fnins.2023.1200370

**Published:** 2023-08-24

**Authors:** Francisco Jiménez-Trejo, Miguel Tapia-Rodríguez, Cristian Arriaga-Canon, Luis A. Herrera, Laura Contreras-Espinosa, Katia Lorena Jiménez-García

**Affiliations:** ^1^Instituto Nacional de Pediatría, Ciudad de México, Mexico; ^2^Instituto de Investigaciones Biomédicas, Universidad Nacional Autónoma de México, Ciudad de México, Mexico; ^3^Instituto Nacional de Cancerología, Ciudad de México, Mexico; ^4^Tecnológico de Monterrey, Escuela de Medicina y Ciencias de la Salud, Ciudad de México, Mexico; ^5^Facultad de Medicina, Universidad Nacional Autónoma de México, Ciudad de México, Mexico

**Keywords:** serotoninomics, serotonin, omics, laboratory techniques, research

## Abstract

Surely, Vittorio Erspamer, discoverer of Enteramine in 1935, and Irvine Page, Maurice M. Rapport and Arda Green, discoverers of Serotonin in 1948, never imagined the biological importance that this fundamental molecule has in the living beings of our planet; from its physiological, passing through endocrine, neural, developmental and reproductive functions and even its role in evolution. For this reason, our workgroup is commemorating these researchers and celebrating their great discovery, which deeply influenced science and medicine, in the present perspective article. As a consequence of their seminal work, and the work of many other researchers in the field of serotonin over the following years, now we stand in front of the practical concept of “Serotoninomics,” which we think will contribute to find out precise answers regarding basic, clinical, and translational research related to serotonin, just as the emerging medical and “omics” sciences have done before.

## Introduction

Since the initial discovery of the 5-hydroxytryptamine (5-HT; C_10_H_12_N_2_O) molecule, first by Vialli and Erspamer (1937), and then by Rapport et al. (1948) in the first half of the past century, we are continuously amazed by the multiple physiological functions in which a role for this indoleamine has been discovered as well for the broad range of species belonging to different biological taxa in which it acts. To date, its involvement in different signaling pathways (for example, in cascades of second messengers or molecular regulations induced by different serotonin receptors), continues to be described, as well as its role in several diseases or pathologies (Adayev et al., 2005).

In 2015, our workgroup proposed the Serotoninomics concept (Jiménez-Trejo and Tapia-Rodríguez, 2015), which encompasses all studies carried out exclusively on serotonin and its system, including the experimental techniques and laboratory tools from the past century and the current one; in Table 1 we enlist the main methodologies that have been used or adapted as study tools for the field of serotonin in the past years; combining these strategies with emerging ones supported in the technological advance of laboratory instruments will lead to a better understanding of the different roles of the serotonergic system in the broad range of biological systems in which it is present.

**Table 1:** Methodologies that have been used or adapted as study tools for the field of serotonin in past years and in the present.

(1) Histochemistry for indolamines or Falck-Hillarp method
(2) Brightfield immunocytochemistry and immunohistochemistry or direct or indirect immunofluorescence, single or multiplex
(3) Brightfield and fluorescence *in situ* hybridization
(4) Super resolution microscopy
(5) Electron microscopy and cryoelectron microscopy
(6) Fluorescence spectroscopy
(7) Flow cytometry
(8) PCR and RT-PCR
(9) Molecular genotyping
(10) Enzyme-linked immunosorbent assay (ELISA)
(11) Optogenetics
(12) Transgenic models
(13) Behavioral trials (using transgenic models, agonists and/or antagonists or other molecules acting over serotonergic pathway elements)
(14) Cell culture and 3D printed models
(15) Chromatography
(16) Western blotting
(17) 2D-gel electrophoresis
(18) CRISPR gene editing
(19) Sanger or high-performance sequencing
(20) Single-cell transcriptomic profiling
(21) Bulk-tissue RNA sequencing and single-cell RNA sequencing
(22) Electrophysiology
(23) X-ray crystallography
(24) Mass spectrometry
(25) Drug delivery via nanoparticles
(26) Genomic analysis
(27) Meta-analysis
(28) Bioinformatics

As could be noted above, a broad range of experimental designs have allow us to locate, detect, analyze and describe the serotonergic systems and their role in different organs, tissues and systems along the past 74 years (Berger et al., 2009); based in this fact, here we request to the International Society for Serotonin Research (ISSR; formerly Serotonin Club),^1^ to standardize and disseminate the idea of this concept related to this monoamine.

Future serotoninomic studies should focus on the description of the different actions carried out not only for the seven receptor families that have been described for serotonin, but also for transporters (membranal: 5HT_T_ and vesicular: VMAT1, VMAT2) and enzymes that synthesize (TPH1, TPH2) or catabolize this indolamine (MAOA, MAOB) in a more integrative way; the analysis and study of serotonin actions at different levels of structure and function of cells, tissues or even in complete organisms, will allow us to achieve a better understanding of the role of 5-HT in health or disease of humans beings and animals (Gaspar et al., 2003; Mohammad-Zadeh et al., 2008). The use of animal models for translational, basic, or clinical studies, using less invasive techniques and protocols, will continue to be a trend along this decade; for the field of serotoninomics a lot of effort must be done in the design and analysis of rigorous and reproducible experiments in the scenario of novel and less invasive experimental techniques; particularly, we think that the study of the relationships of serotonergic system with the gastrointestinal tract microbiota will be of particular interest when it could be done with such scope, because of the current difficulty of resembling in a cell culture a complex environment such as that present in the gastrointestinal tract—in which the largest amount of 5-HT is produced—; it is an exciting possibility that local interactions in the different subpopulations of microbiota could be modulated by this indoleamine but further, effective experimental designs must be realized to be able to test this idea.

The study of serotonergic system components in the nervous system of mammals currently remains as a methodological challenge because of the physiological nature of this indoleamine as a neurotransmitter (Zhao and Piatkevich, 2023); however, stronger efforts must be done to achieve molecular, pharmacological treatments, long lasting and without secondary effects if we want to get a cure for the neuropsychiatric disorders, such as anxiety and depression in which serotonergic system is related, and to further understand how different drugs (SSRIs, agonist, antagonist and psychedelics) are participating in mental illness and addiction processes to be able to restore the mental homeostasis of such patients; the use of animal models and genetically encoded serotonergic sensors, and the use of non-invasive imaging techniques are promising approaches which will gain importance in the years to come.

Next, we want to highlight the relevant role that serotoninomics will have within the field of Mammalian and Human Reproductive Biology; it is well-known that 5-HT participates in several reproductive processes, ranging from male sexual behavior to the regulation of androgen production (steroidogenesis) via a cAMP-dependent pathway in an autocrine way within Leydig cells, which in turn could promote sperm maturation and probably fertilization. In addition, serotonin has been found to play an important role in reproductive diseases such as varicocele, infertility, low libido or abnormal sexual behavior, and testicular carcinoid and prostate cancer. Particularly, our research group has described local systems for the synthesis and degradation of serotonin in the testis, epididymis, and spermatozoa (Jiménez-Trejo et al., 2021). Furthermore, we have detected that the spermatogenic stem cells type A (As SSC)—primitive cells that give rise to spermatozoa—also contain it (data to be published); overall we think that these important topics need to be further analyzed in the upcoming years by future generations of scientists around the world.

The involvement of serotoninomics in biological processes is extensive and it is clear that a lot of research must to be done to further expand its physiological significance and to achieve a more complete understanding about its role in the pathologies in which its balance has been altered (for example, female infertility, andrology, embryology, animal biotechnology, stem cell biology, transplant technology or regenerative medicine, oncofertility and cancer, molecular pharmacology; Horgan and Kenny, 2011; Barh et al., 2013). Training about recent technological innovations, such as spectral flow cytometry, cryo-electron microscopy, focused ion beam microscopy, correlative microscopy, expansion microscopy, super-resolution microscopy, integration of multi-omic approaches at a single-cell resolution: Bulk-Single-Cell RNA Sequencing, will allow us to gain new perspectives into the function and cell biology and disease, which in turn will enable us to perform better therapeutic interventions (Hasin et al., 2017; Chakraborty et al., 2018; Hegenbarth et al., 2022). As mentioned by the organizers of the Congress of Innovations in Reproductive Medicine 2030: IVF Technologies (RM2030; November 16–18, 2023, Paris, France): “The progress of medicine depends on new technologies and revolutionary concepts, which are translated into practice to solve long-standing problems for the benefit of patients, as well as fostering communication and debate of cutting-edge ideas among thoughtful selected leaders.”

Finally, the pioneers in the field of serotonin probably never imagined the extent of the involvement of this indoleamine in life sciences. For this reason, today we commemorate and celebrate their success, which influenced the good practices of medicine and science as a whole. For the years to come, we believe that serotoninomics, contributing with other omics areas, will increase our understanding of the biological processes, ranging from molecular level to the whole, complex organisms.

## Data availability statement

The raw data supporting the conclusions of this article will be made available by the authors, without undue reservation.

## Author contributions

All authors listed have made a substantial, direct, and intellectual contribution to the work and approved it for publication.

## Conflict of interest

The authors declare that the research was conducted in the absence of any commercial or financial relationships that could be construed as a potential conflict of interest.

## Publisher’s note

All claims expressed in this article are solely those of the authors and do not necessarily represent those of their affiliated organizations, or those of the publisher, the editors and the reviewers. Any product that may be evaluated in this article, or claim that may be made by its manufacturer, is not guaranteed or endorsed by the publisher.

## Footnotes

^1^ISSR is an international non-profit professional association for biomedical scientists interested in any aspect of serotonin research. This society was founded in 1987 and currently has about 350 members around the world. The ISSR organizes an international meeting every 2 years with symposia and short talks that cover all aspects of serotonin research; in addition, its provides financial support to young scientists who wish to attend these meetings; in the present year (2023), the Society organized its 20th International Meeting, called “Serotonin in the Mexican Caribbean,” which was held in Cancun, Mexico from April 23–27. Furthermore, the ISSR organizes annual networking events in association within the annual meetings of the Society for Neuroscience (SfN) and the Federation of European Neuroscience Societies (FENS) Information taken from: www.serotoninclub.org/about#
